# Capsaicin: a spicy way in liver disease

**DOI:** 10.3389/fphar.2024.1451084

**Published:** 2024-08-30

**Authors:** Shenghao Li, Liyuan Hao, Fei Yu, Na Li, Jiali Deng, Junli Zhang, Shuai Xiong, Xiaoyu Hu

**Affiliations:** ^1^ School of Clinical Medicine, Chengdu University of Traditional Chinese Medicine, Chengdu, China; ^2^ Department of Infectious Diseases, Hospital of Chengdu University of Traditional Chinese Medicine, Chengdu, China; ^3^ Jiangsu Province Hospital of Chinese Medicine, Nanjing, China

**Keywords:** capsaicin, liver injury, NAFLD, liver fibrosis, liver cancer

## Abstract

The incidence of liver disease continues to rise, encompassing a spectrum from simple steatosis or non-alcoholic fatty liver disease (NAFLD) to non-alcoholic steatohepatitis (NASH), cirrhosis and liver cancer. Dietary habits in individuals with liver disease may significantly impact the treatment and prevention of these conditions. This article examines the role of chili peppers, a common dietary component, in this context, focusing on capsaicin, the active ingredient in chili peppers. Capsaicin is an agonist of the transient receptor potential vanilloid subfamily 1 (TRPV1) and has been shown to exert protective effects on liver diseases, including liver injury, NAFLD, liver fibrosis and liver cancer. These protective effects are attributed to capsaicin’s anti-oxidant, anti-inflammatory, anti-steatosis and anti-fibrosis effects. This article reviewed the different molecular mechanisms of the protective effect of capsaicin on liver diseases.

## 1 Introduction

The liver plays a vital role in energy metabolism, bile acid secretion, drug metabolism, detoxification, among other functions (Luo et al., 2022). Liver disease causes 2 million deaths each year, accounting for 4 percent of all deaths, and about two-thirds of liver-related deaths occur in men (Devarbhavi et al., 2023). The incidence of liver disease continues to increase (Yu Y. et al., 2014), encompassing conditions ranging from simple steatosis and non-alcoholic fatty liver disease (NAFLD) to non-alcoholic steatohepatitis (NASH), cirrhosis and liver cancer (Li et al., 2019). Therefore, it is crucial to find effective means to prevent the occurrence of liver diseases.

In recent years, capsaicin has attracted attention for its potential in the prevention and treatment of many diseases (Radhakrishna et al., 2024). Capsaicin (trans-8-methyl-N-vanillyl-6-nonenamide), a naturally occurring alkaloid, is the active component in Capsicum plants and serves as an agonist of transient receptor potential vanilloid subfamily 1 (TRPV1). This spicy substance in red chili peppers features a long hydrophobic carbon end with a polar amide group and a benzene ring (Li et al., 2020). Capsaicin exhibits numerous beneficial properties, including protection against liver damage (Fukuta et al., 2020), anti-diabetic (Wang et al., 2012), anti-obesity (Li et al., 2020), anti-liver fibrosis (Sheng et al., 2020), anti-liver cancer (Ates et al., 2022), relieve pain (Caprodossi et al., 2011) and anti-oxidant (Chen et al., 2015) and so on. This article reviewed the protective effects of capsaicin on liver diseases through various mechanisms of action. A better understanding of the specific role of capsaicin in liver pathogenesis may provide new directions for the treatment and prevention of liver diseases.

## 2 Capsaicin

Capsaicin (trans-8-methyl-N-vanillyl-6-nonenamide) is the main compound responsible for the spicy flavor of Capsicum plants (Santos et al., 2023). It is insoluble in water and contains a vanillyl group (the head), an amide group (the neck), and a fatty acid chain (the tail) (Musolino et al., 2024). Capsaicin functions through both TRPV1-dependent and TRPV1-independent pathways, with evidence suggesting that its biological effects may be mediated by either mechanism (Sánchez et al., 2022; Zhang et al., 2020). Capsaicin is a high affinity agonist for the TRPV1 (Li et al., 2021). The affinity between capsaicin and TRPV1 channels is highly selective and potent. TRPV1 is a non-selective cation channel that responses to pH, temperature, and endogenous lipids (Alawi and Keeble, 2010). It is activated directly or indirectly by various neuroinflammatory mediators and endogenous inflammatory mediators, such as calcitonin gene-related peptide (CGRP) or substance P (SP). TRPV1 is also involved in various physiological and pathological processes in the body, including cough, pain, inflammation, hearing, taste, gastrointestinal movement, blood pressure regulation, apoptosis, fat metabolism, pruritus and tumor pathologic processes (Li and Wang, 2021). Activation of TRPV1 led to the opening of Ca^2+^ channels, an influx of Ca^2+^, and increased Ca^2+^ concentration in the cytoplasm, thus promoting the release of neuropeptides, vasoactive intestinal peptides and excitatory amino acids by neurons and their fibers. This depletes and inhibits their formation, and blocking the pain conduction pathway from peripheral nerve to central nerve (Sharma et al., 2013). After activating TRPV1, capsaicin induces pain afferent neurons to release SP. Continuous activation leads to SP exhaustion, eventually preventing the perception and transmission of pain, thus exerting a pain-relieving effect (Caprodossi et al., 2011). While capsaicin exerts many of its effects through TRPV1 activation, some studies have reported capsaicin-induced effects that occur independently of TRPV1. In addition to activating TRPV1, capsaicin also regulates the production of reactive oxygen species (ROS) (Wu et al., 2022), the flux of other ions across cell membranes (Reilly et al., 2012), and the fluidity of cell membranes, impacting various cellular functions (Prakash and Srinivasan, 2010). Consequently, it has also been extensively studied as a powerful anti-oxidant and anti-inflammatory agent (Braga Ferreira et al., 2020). Capsaicin alleviates inflammatory responses and the Warburg effect in a TRPV1-independent manner by targeting PKM2, LDHA and COX-2 (Zhang Q. et al., 2022). Moreover, capsaicin induces AMPK and p53 activation and triggers cell death in a TRPV1-independent manner (Bao et al., 2019). Overall, capsaicin’s ability to act through both TRPV1-dependent and independent pathways highlights its therapeutic potential. This dual action allows capsaicin to modulate a wide range of biological processes, making it a valuable compound for further research and clinical applications.

## 3 Liver injury and capsaicin

### 3.1 Liver injury

Liver injury is a major threat to human health worldwide, with causes including viral hepatitis, autoimmune liver disease, liver ischemia, and drug toxicity (Stravitz and Lee, 2019). The recent increase in the use of newly developed drugs and herbal or dietary supplements has increased the risk of liver damage. Drug-induced liver injury (DILI) is a rare but significant condition that can appear after exposure to various drugs, herbs, and dietary supplements. The severity of DILI varies, and severe liver damage can progress to acute liver failure, potentially resulting in death within days of onset or liver transplantation (Hassan and Fontana, 2019). Excessive alcohol consumption is another major cause of liver damage and liver failure globally (Koneru et al., 2018). The rapid progression of alcoholic liver disease (ALD) lead to liver fibrosis and cirrhosis. Alcohol metabolism produces toxic metabolites that cause tissue and organ damage through an inflammatory cascade involving numerous cytokines, chemokines, and ROS (Dukić et al., 2023). Currently, effective strategies for treating liver injury are lacking. Therefore, there is an urgent need to develop new therapeutic agents to inhibit liver damage and reduce the risk of severe liver failure in affected patients.

### 3.2 Roles of capsaicin in the treatment of liver injury

#### 3.2.1 Drug-induced liver injury

Capsaicin has demonstrated a protective effect on liver injury (Table 1). Studies have shown that carbon tetrachloride (CCl4) can significantly increase the levels of aspartate aminotransferase (AST) and alanine aminotransferase (ALT) in the rat model. However, the combined of liposomes encapsulating astaxanthin (Asx)-R (Asx-R-Lipo) and liposomes encapsulating capsaicin (Cap) (Cap-Lipo) significantly reduced CCL4-induced elevation of AST and ALT (Fukuta et al., 2020). Additionally, both the Asx-R-Lipo and Cap-Lipo treatment groups showed a reduction in ALT levels, with Cap-Lipo exhibiting a more pronounced decrease. Capsaicin has also shown a protective effect against N-acetyl-para-aminophenol (APAP)-induced acute liver injury (ALI). This beneficial effect might be attributed to capsaicin’s ability to inhibit the high mobility group box 1 (HMGB1)/toll-like receptor 4 (TLR4)/nuclear factor κB (NF-κB) signaling pathway, reduce the release of pro-inflammatory cytokines, diminish hepatic oxidative stress induced by APAP and alleviate hepatocyte apoptosis (Zhan et al., 2020). Cyclophosphamide disrupts the anti-oxidant system by producing ROS and led to liver injury. Capsaicin’s hepatoprotective effect in this context is due to its ability to reduce ROS production, inhibit inflammation and suppress the expression of apoptosis protein Caspase-3 (Alam et al., 2023). Moreover, studies have also demonstrated that capsaicin, when used in combination with other therapeutic approaches, enhances its protective effects against liver diseases. Capsaicin and cannabinoids improved liver pathology and liver function following thioacetamide-induced acute injury in mice (Avraham et al., 2008).

**TABLE 1:** Effects of capsaicin on liver injury.

Model	Capsaicin dosage	Main functions	Reference
CCl4-induced liver injury model rat	0.5 µmol Asx/kg and 1 µmol capsaicin/kg	↓ALT, AST	Fukuta et al. (2020)
Alcohol-induced acute liver injury in mice	10 and 20 mg/kg	↓ALT, AST	Koneru et al. (2018)
Acetaminophen-induced acute liver injury in mice	1 mg/kg	↓IL-6, IL-1β, TNF-α, MDA; ↑SOD, GSH	Zhan et al. (2020)
Cyclophosphamide-induced liver injury in rat	10 mg and 20 mg/kg	↓serum liver markers (AST, ALT, ALP, BLI), IL-1β, TNFα, caspase-3	Alam et al. (2023)
Thioacetamide-induced acute injury in mice	1.25 μg/kg	↓AST, ALT, ammonia, and bilirubin	Avraham et al. (2008)
Septic acute liver injury in mice	5 or 20 mg/kg	↓ALT, AST, MDA, ROS, NF-kB, TLR4, IL-1β, TNF-α, caspase 3 ↑sirtuin1, Nrf2, SOD, HO-1	Ghorbanpour et al. (2023)
Alcohol-induced liver injury in mice	0.014%	↑body, liver, and brain weights	Pyun et al. (2014)

#### 3.2.2 Sepsis-induced acute liver injury

Capsaicin is known for its anti-oxidant and anti-inflammatory effects. It has demonstrated beneficial effects on apoptosis and mitochondrial function in acute liver injury (ALI) associated with sepsis. High doses of capsaicin have been shown to reduce serum levels of ALT and AST, reverse and/or improved the expression of apoptosis-related proteins, and regulate mitochondrial and metabolic regulators, as well as inflammation-related molecules. These findings suggest that capsaicin protects the liver during ALI in sepsis, likely due to its ability to downregulate oxidation and inflammatory processes and potentially alleviate mitochondrial dysfunction and apoptosis (Ghorbanpour et al., 2023).

#### 3.2.3 Alcoholic liver injury

Studies have shown that a diet rich in capsaicin can be used as an adjunct treatment for liver damage or disease caused by alcohol consumption (Koneru et al., 2018; Pyun et al., 2014). In an earlier study, it has shown that a diet containing capsaicin reduces acute ethanol-induced lipid accumulation in the liver of rats (Sambaiah and Satyanarayana, 1989). Capsaicin inhibits Cytochrome P450 2E1 (CYP2E1) and reduces ROS production. This inhibition lead to a decrease in free radical formation and oxidative stress, restoration of the MMP/TIMP balance, reducing liver injury (Koneru et al., 2018). Furthermore, studies have also demonstrated that capsaicin could be used in combination with dietary modifications enhances its protective effects against liver diseases. Dietary curcumin and capsaicin has been shown to prevent loss of alcohol-induced body, liver, and brain weights and inhibit alcohol-induced oxidative stress in BALB/c mice (Pyun et al., 2014).

## 4 Non-alcoholic fatty liver disease and capsaicin

### 4.1 Non-alcoholic fatty liver disease

NAFLD affects 25% of the world’s population and is the most prevalent liver disease. It presents a diverse phenotype, ranging from simple steatosis to NASH, fibrosis, cirrhosis, and hepatocellular carcinoma (HCC) (Diehl and Day, 2017). NAFLD is a complex systemic disease characterized by liver lipid accumulation, lipotoxicity, insulin resistance, intestinal dysbiosis, and inflammation (Tilg et al., 2021). The primary driver of NAFLD is excess nutrition, leading to the expansion of the fat pool and the accumulation of ectopic fat (Kragh Petersen et al., 2020). In this case, macrophage infiltration in the visceral adipose tissue produces a pro-inflammatory state that promotes insulin resistance. In insulin resistance, inappropriate lipolysis results in the continuous delivery of fatty acids to the liver, which, along with increased *de novo* lipogenesis, disrupts liver metabolism (Powell et al., 2021). Imbalances in lipid metabolism lead to the formation of lipotoxic lipids, promoting oxidative stress, inflammasome activation and apoptosis, and subsequently stimulate inflammation, tissue regeneration and fibrosis (Friedman et al., 2018; Sanyal, 2019). The multiple parallel hits theory explained the progression of NAFLD (Tilg and Moschen, 2010). Multiple hits induced adipokine secretion, oxidative stress at the endoplasmic reticulum and cellular levels, and subsequently induced hepatic steatosis (Takaki et al., 2013). This phenomenon made the liver vulnerable to multiple effects, including inflammation, mitochondrial dysfunction, lipocytokine imbalances, apoptosis dysregulation, oxidative stress, intestinal dysbiosis, HSCs activation, and production of pro-fibrotic factors, ultimately leading to NASH and cirrhosis (Takai and Jin, 2018). Studies have shown that capsaicin may have a role in improving NAFLD. Capsaicin has been found to reduce liver lipid accumulation, mitigate oxidative stress, and decrease inflammation. These effects suggest that capsaicin could be a potential therapeutic agent for managing NAFLD and its progression to more severe liver diseases.

### 4.2 Roles of capsaicin in the treatment of non-alcoholic fatty liver disease

#### 4.2.1 Anti-steatosis

Capsaicin exhibits anti-steatosis effect (Table 2). It reduces lipid accumulation and also decreased glucose and fatty acid uptake in HepG2 (Hochkogler et al., 2018). Studies have shown that capsaicin and hesperidin prevent hepatic steatosis and other metabolic syndrome-related changes in rats fed a western diet (Mosqueda-Solís et al., 2018a). Additionally, topical capsaicin cream combined with moderate exercise has been shown to prevent hepatic steatosis, dyslipidemia and elevated blood pressure in hypoestrogenic obese rats (de Lourdes Medina-Contreras et al., 2020). Dietary capsaicin reduces liver steatosis and insulin resistance in obese mice fed a high-fat diet (HFD) (Kang et al., 2010). Capsaicin has been shown to improve lipid metabolism in the liver (Kang et al., 2011). It stimulates the expression of carnitine palmitoyl transferase (CPT)-1 and CD36, enzymes involved in β-oxidation and hepatic fatty acid inflow. Conversely, capsaicin decreases the expression of key enzymes involved in fatty acid synthesis, such as acetyl Co-A carboxylase (ACC) and fatty acid synthase (FAS) (Shin et al., 2020). These changes suggest that capsaicin not only helps in reducing fat accumulation but also improves overall lipid metabolism in the liver, making it a potential therapeutic agent for managing hepatic steatosis and related metabolic disorders.

**TABLE 2:** Effects of capsaicin on non-alcoholic fatty liver disease.

Model	Capsaicin dosage	Main functions	Reference
3T3-L1 and HepG2 cells	3T3-L1:6.91%; HepG2:100 μM	↓mitochondrial oxygen consumption and reduced glucose and fatty acid uptake	Hochkogler et al. (2018)
Western diet in rats	4 mg/kg	↓hepatic lipid accumulation	Mosqueda-Solís et al. (2018a)
High fat diet in mice	0.015%	↑PPAR-α	Kang et al. (2010)
High fat diet in KKAy mice	0.015%	↓fasting glucose/insulin and triglyceride, inflammatory adipocytokine genes	Kang et al. (2011)
High-fat diet in mice	0.075%	↑CPT-1 and CD36 ↓ACC and FAS	Shin et al. (2020)
High-fat diet in mice	capsaicin 0.4 mg/kg, menthol 20 mg/kg, and cinnamaldehyde 2 mg/kg	↓weight gain, lipid accumulation and insulin resistance ↑brown adipose tissue activation	Kaur et al. (2022)
High-fat diet in mice	2 mg/kg	↓weight gain and food intake, triglyceride, cholesterol, glucose, and insulin levels	Wang et al. (2020)
High-fat diet in mice	2 mg/kg	↑BAT associated genes	Baboota et al. (2014a)
3T3-L1 cells and mice	3T3-L1 cells:0.1–100 μM; mice:2 mg/kg		Baboota et al. (2014b)
HepG2 cells	200 and 300 µM	↑AMPK and PGC-1α	Bort et al. (2019a)
SD rats	30 mg/kg	↓lipid accumulation	Wu et al. (2017)
HepG2 cells	200 μM	↑ROS and AMPK	Bort et al. (2019b)
Caco-2 cells	0.1–100 µM	↑FATP2, FATP4, IFABP, CD36, PPARα and PPARγ	Rohm et al. (2015)
Western diet in rats	4 mg/kg	↑UCP1 and CIDEA	Mosqueda-Solís et al. (2018b)
High-fat diet in rats	10 mg/kg	↓vimentin, peroxiredoxins, and NQO1	Joo et al. (2010)
High-fat diet in rats	bean powder (15%) plus capsaicin (0.015%)	↓hepatic cholesterol and triglycerides	Pande and Srinivasan (2012)
High-fat diet in rats	HCCMS, 3,382 mg/kg/d (containing 30 mg capsaicin); M-CCMS, 1,128 mg/kg/d (10 mg capsaicin); L-CCMS, 367 mg/kg/d (3 mg capsaicin)	↑PPARα, PPARγ, UCP2, and adiponectin ↓ leptin	Tan et al. (2014)
High-fat diet in rats	0.15 g capsaicin/kg and/or 1.5 g curcumin/kg	↓hepatic fat accumulation and leptin	Seyithanoğlu et al. (2016)
High-fat diet in mice	0.015%	↑PPAR-α	Hu et al. (2017)
High-fat diet in mice	0.010%	↑Muc2 and Reg3g	Shen et al. (2017)
High-fat diet in mice	16 mg/kg	↑adiponectin ↓leptin, free fatty acid and insulin concentrations	Shanmugham and Subban (2022)
Overweight women	125 mg green tea, 25 mg capsaicin and 50 mg ginger extracts	↓serum insulin concentrations; ↑quantitative insulin sensitivity check index and GSH	Taghizadeh et al. (2017)

#### 4.2.2 Anti-obesity

The anti-obesity effect of capsaicin has been confirmed through various models, ranging from cells to animals and humans (Li et al., 2020). Capsaicin promotes weight loss by increasing energy expenditure and increasing satiety (Elmas and Gezer, 2022), inhibiting the production of white adipose tissue (WAT) and activating the activity of brown adipose tissue (BAT) (Kaur et al., 2022). It also improves the gut microbiota (Wang et al., 2020; Baboota et al., 2014a) and other pathways mediated.

In 3T3-L1 cells, capsaicin inhibits adipocyte differentiation by activating TRPV1, which induces the browning of white adipocyte, increases heat production, and decreases intracellular lipid content (Baboota et al., 2014b). Activation of TRPV1 enhances peroxisome proliferator-activated receptor gamma (PPAR-γ) expression and deacetylation, promoting the browning of white adipose tissue (Krishnan et al., 2019). Capsaicin reduces lipid accumulation and glucose and fatty acid uptake in 3T3-L1 cells (Hochkogler et al., 2018). Capsaicin activates AMP-activated protein kinase (AMPK) and inhibits the protein kinase B (AKT)/mammalian target of rapamycin (mTOR) pathway, a major regulator of liver adipogenesis. In addition, capsaicin blocks autophagy and increases peroxisome proliferator-activated receptor gamma coactivator-1A (PGC-1a) protein, suggesting that capsaicin acts as an anti-lipogenic compound in HepG2 cells (Bort et al., 2019a). Studies have indicated that triglyceride content and lipid droplets in hepatocytes are significantly reduced by capsaicin, highlighting its potential to inhibit lipid production in HepG2 cells (Wu et al., 2017). Additionally, capsaicin decreases basal neutral lipid content and increases TRPV1 levels by activating AMPK and PPAR-γ pathways in HepG2 cells (Bort et al., 2019b).

In rats, capsaicin reduces body weight, inhibits fat accumulation and induces heat production (Ludy et al., 2012). Oral administration of capsaicin for 5 weeks in HFD-fed rats results in increased UCP1 expression in WAT, along with changes in protein expression related to thermogenesis, lipid metabolism, redox-regulation, signal transduction and energy metabolism (Joo et al., 2010). Capsaicin-loaded nanoemulsion effectively reduces the body weight gain, serum lipid level and adipose tissue mass in obese male SD (Sprague Dawley) rats induced by HFD (Lu et al., 2016). In addition, capsaicin reduces weight gain and lowered triglyceride levels in HFD-fed rats without affecting feed intake (Pande and Srinivasan, 2012). Capsaicin-chitosan microspheres (CCMSs) can regulate body weight, body mass index, organ index, body fat, fat-to-weight ratio, and blood lipid levels (Tan et al., 2014). Studies have also demonstrated that capsaicin could be used in combination with other treatments or dietary modifications to enhance its protective effects against liver diseases. In rats fed a western diet, capsaicin alone or in combination with hesperidin reduces adipocyte size and induces the browning of WAT and reduces weight gain by upregulating UCP1 and PRDM16 (Mosqueda-Solís et al., 2018b). Additionally, capsaicin inhibits the histological features of NAFLD by decreasing hepatic fat accumulation and increasing leptin levels associated with inflammation (Seyithanoğlu et al., 2016). Moreover, dietary curcumin and capsaicin treatment reduced weight gain and liver lipid levels induced by HFD consumption (Seyithanoğlu et al., 2016). These findings suggest that capsaicin has significant potential in managing obesity and NAFLD through various mechanisms, including promoting the browning of WAT, enhancing thermogenesis, and improving lipid metabolism.

The application of capsaicin has been shown to reduce liver fat in mice fed a HFD. Capsaicin stimulates the expression of CPT-1 and CD36, while decreases the expression of key enzymes involved in fatty acid synthesis, such as acetyl Co-A carboxylase (ACC) and fatty acid synthase (FAS). Additionally, capsaicin treatment increases adiponectin levels in liver tissues. These results suggest that capsaicin inhibits liver fat accumulation in mice by upregulating β-oxidation and *de novo* lipogenesis in HFD-induced NAFLD mice (Shin et al., 2020). Studies have shown that antibiotics treatment significantly reduces intestinal inflammation and leakage caused by HFD. Diet capsaicin increases the expression of PPAR-α in adipose tissue. Animals treated with both capsaicin and antibiotics showed the least weight gain and had the smallest fat pad index. Their livers exhibited the lowest levels of fat accumulation, and this combination therapy also resulted in the highest insulin responsiveness (Hu et al., 2017). Regardless of whether the TRPV1 channel was activated, capsaicin reduced food intake and demonstrated anti-obesity effects, which were mediated by changes in gut microbiota and concentrations of short-chain fatty acids (SCFAs) (Wang et al., 2020). One study has shown that the anti-obesity effects of capsaicin in HFD-fed mice are associated with an increase in the population of gut bacteria *Akkermansia muciniphila* (Shen et al., 2017). Capsanthin-enriched pellets and capsaicin pellets effectively reduced body weight in mice. Treatment with capsanthin-enriched pellets resulted in a 37.0% reduction in inguinal adipose tissue and a 43.64% reduction in epididymal adipose tissue (Shanmugham and Subban, 2022). Capsaicin exhibited an antagonistic effect on HFD-induced obesity in mice without reducing energy intake (Baskaran et al., 2017).

There is a positive correlation between dietary capsaicin consumption and markers of body obesity and fatty liver (Martínez-Aceviz et al., 2023). A study involving fifteen subjects has shown that diet capsaicin increases feelings of fullness when food intake is not restricted, and after dinner, capsaicin prevents the effects of negative energy balance on appetite (Janssens et al., 2014). Adding capsaicin to the diet has been shown to increase energy expenditure, helping to maintain negative energy balance by counteracting the adverse effects of reduced energy expenditure components (Janssens et al., 2013). Taking dietary supplements containing green Tea, capsaicin and ginger extracts for 8 weeks in overweight women has shown beneficial effects on body weight, body mass index, insulin metabolism markers, and plasma glutathione levels (Taghizadeh et al., 2017). Moreover, studies have shown that a combination of capsaicin, green tea, and CH-19 sweet pepper can reduce body weight in humans by reducing energy intake, suppressing hunger, and increasing satiety (Reinbach et al., 2009). These findings suggest that capsaicin could be used in combination with other treatments or dietary modifications play a valuable role in weight management and metabolic health, contributing to improved insulin metabolism and oxidative stress markers, while also enhancing satiety and energy expenditure.

#### 4.2.3 Improve insulin resistance

HFD or overfeeding can reduce muscle glucose uptake and increase liver gluconeogenesis, leading to insulin resistance. Insulin resistance in the liver and skeletal muscle leads to hyperglycemia, hyperinsulinemia, contributing to dyslipidemia and fatty liver (Czech, 2017). Capsaicin has been shown to have protective effects against NAFLD and metabolic disorders by addressing insulin resistance and hepatic steatosis (Kang et al., 2010). It also has preventive effects on insulin resistance in rats fed a western diet (Mosqueda-Solís et al., 2018a). Capsaicin inhibits sugar absorption in the gut (Zhang et al., 2017), reduces liver gluconeogenesis, increases glycogen synthesis, improves intestinal microbiota and bile acids and enhances insulin resistance (Hui et al., 2019). Nonivamide, a capsaicin analog, promotes insulin signaling, stimulates glucose transporter 2 (GLUT2) transport to the membrane, and improves NAFLD (Wikan et al., 2020). Pelargonic acid vanillylamide (PAVA) alleviates NAFLD by exhibiting anti-inflammatory effects and improving insulin resistance mediated by the Janus kinase 2 (JAK2)/signal transducer and activator of transcription 3 (STAT3) pathway (Wikan et al., 2023). Oral capsaicin attenuates the proliferation and activation of autoreactive T cells in the pancreatic lymph node, protecting mice from the development of type 1 diabetes (Nevius et al., 2012). In type 2 diabetes, dietary capsaicin activation of TRPV1 improved abnormal glucose homeostasis and increases plasma and ileum glucagon-like peptide 1 (GLP-1) levels (Wang et al., 2012). Capsaicin improves glucose tolerance and insulin sensitivity in mice by regulating the gut microbial-bile acid-farnesoid X receptor (FXR) axis (Hui et al., 2019). Studies have suggested that dietary capsaicin reduces fasting blood sugar, insulin, leptin levels and significantly reduces impaired glucose tolerance in obese mice (Kang et al., 2010). It also reduces metabolic disorders in obese/diabetic KKAy mice by increasing the expression of adiponectin and its receptors (Kang et al., 2011). Capsaicin enhances insulin secretion at various stage during hyperglycemic clamp, increases β-cell proliferation and decreases β-cell apoptosis by enhancing insulin/IGF-1 signaling, thereby increasing β-cell mass (Kwon et al., 2013). In addition, regular supplementation of capsaicin improves postprandial hyperglycemia, hyperinsulinemia and fasting lipid metabolism disorders in women with gestational diabetes mellitus (GDM) (Yuan et al., 2016).

In a word, in NAFLD, key molecules and pathways play critical roles in regulating lipid metabolism and disease progression. CPT-1 and CD36 are essential for promoting β-oxidation and hepatic fatty acid uptake, while ACC and FAS are central to fatty acid synthesis. Their expression is tightly controlled, impacting lipid metabolism directly. The activation of AMPK and inhibition of the AKT/mTOR pathway are crucial in suppressing hepatic adipogenesis. Increased PGC-1α levels and autophagy blockade further support hepatic lipid homeostasis. UCP1 and PRDM16 contribute to NAFLD inhibition by inducing the browning of WAT and reducing adipocyte size. The gut microbial-bile acid-FXR axis plays a significant role in enhancing glucose tolerance and insulin sensitivity, while the insulin/IGF-1 signaling pathway increases β-cell mass, further modulating glucose and lipid metabolism.

Capsaicin improves hepatic lipid metabolism by upregulating CPT-1 and CD36 and downregulating ACC and FAS, thus promoting β-oxidation and reducing fatty acid synthesis. This effect is mediated through AMPK activation and AKT/mTOR inhibition, key regulators of liver adipogenesis. Capsaicin also blocks autophagy, elevates PGC-1α levels, reduces adipocyte size, and induces WAT browning by upregulating UCP1 and PRDM16. Additionally, capsaicin improves glucose tolerance and insulin sensitivity via modulation of the gut microbial-bile acid-FXR axis, and enhances β-cell function by increasing proliferation and reducing apoptosis through insulin/IGF-1 signaling, thereby augmenting β-cell mass.

## 5 Liver fibrosis and capsaicin

### 5.1 Liver fibrosis

Liver fibrosis and end-stage cirrhosis are common consequences of all major chronic liver diseases, with HSCs activation being the primary mechanism underlying the deposition of fibrotic tissue (Elpek, 2014). Fibrosis serves as a wound-healing defense mechanism triggered by inflammation or injury. However, the immune system’s destruction of organ structures and inherent inflammation in the liver lead to immune deficiency and immune paralysis. Liver fibrosis is characterized by extracellular matrix deposition and persistent loss of the tissues that perform liver function (Wang et al., 2023). If left untreated, liver fibrosis can progress to cirrhosis, HCC and eventually liver failure (Cheng et al., 2021). The pathophysiology of liver fibrosis is multifactorial, with the activation of HSCs driving its development. When activated, HSCs are associated with fibrotic matrix deposition and fibrous collagen production (Neshat et al., 2021). Unfortunately, there is currently no effective treatment for liver fibrosis other than liver transplantation (Wang et al., 2023; Cheng et al., 2021).

### 5.2 Roles of capsaicin in the treatment of liver fibrosis

Liver fibrosis caused by the activation of HSCs is associated with the incidence of liver diseases (Zhang WS. et al., 2022). Previous studies have supported capsaicin’s inhibitory effect on HSCs, demonstrating its important role in mitigating liver fibrosis (Table 3). Capsaicin inhibits M1 macrophage polarization by targeting Notch signaling, resulting in decreased secretion of the inflammatory factor TNF-α, which weakens myofibroblast regeneration and fibrosis formation of HSCs (Sheng et al., 2020). Capsaicin inhibits dimethylnitrosamine (DMN)-induced hepatotoxicity, NF-κB activation, and collagen accumulation. Specifically, capsaicin reduces the increase of α-SMA, collagen type I, MMP-2 and TNF-α. In hematopoietic stem cells, capsaicin inhibits TGF-β1-induced increased expression of α-SMA and collagen type I by activating PPAR-γ. These results suggest that capsaicin improves liver fibrosis by inhibiting the TGF-β1/Smad pathway through PPAR-γ activation (Choi et al., 2017). The inhibitory effect of dietary capsaicin on liver fibrosis *in vivo* has been confirmed using two well-established mouse models of liver fibrosis: bile duct ligation (BDL) and CCl4. This is demonstrated by reduced fibrosis related damage, reduced collagen deposition and α-smooth muscle actin (αSMA)^+^ cells, and reduced expression of profibrogenic markers in isolated HSCs (Bitencourt et al., 2015). Capsaicin also inhibits cell proliferation, reduces cell activation, and reduces hydrogen peroxide production, lowers levels of tissue inhibitor of metalloproteinases-1 (TIMP-1) and transforming growth faction-1 (TGF-1). Consequently, capsaicin effectively reduces the degree of liver fibrosis, inhibits the proliferation of HSCs, and promotes cell apoptosis (Yu FX. et al., 2014).

**TABLE 3:** Effects of capsaicin on liver fibrosis.

Model	Capsaicin dosage	Main functions	Reference
CCl4-induced liver fibrosis in mice	0, 2.5 and 5 mg/kg	↓TNF-α	Sheng et al. (2020)
DMN-induced liver fibrosis in rats	0.5 and 1.0 mg/kg	↓α-SMA, collagen type I, MMP-2, and TNF-α, TGF-β1 ↑Smad7	Choi et al. (2017)
CCl4-induced fibrosis in mice	0.01%	↓Col1a1, αSMA and Loxl2	Bitencourt et al. (2015)
CCl4-induced fibrosis in rats	0, 2.5, 5.0 and 7.5 mg/kg	↓TIMP-1, TGF-1, Bcl-2 ↑Bax, cyto c, caspase-3	Yu et al. (2014b)

## 6 Liver cancer and capsaicin

### 6.1 Liver cancer

Liver cancer is one of the most common malignancies and the third leading cause of cancer-related death worldwide (Sung et al., 2021). The common types of liver cancer include HCC, cholangiocarcinoma (CC), and hepatocellular cholangiocarcinoma (HCC/CC) (Zhang and Zhou, 2019). Liver cancer exhibits high incidence and mortality rates, and traditional treatments, such as transarterial chemoembolization (TACE) or sorafenib, have significant limitations, including cancer recurrence, drug ineffectiveness, and adverse reactions (Kim et al., 2022). Natural products have shown promising anti-liver cancer properties, anti-oxidation, induction of apoptosis, inhibition of cancer cell proliferation and inhibition of angiogenesis (Diab et al., 2022; Guo et al., 2019; Waziri et al., 2018). The pathogenesis of HCC is complex, involving processes such as abnormal cell and tissue regeneration, angiogenesis, genomic instability, cell proliferation and alterations in signal pathway. Studies have found that capsaicin plays a role in various stages of liver cancer progression (Table 4).

**TABLE 4:** Effects of capsaicin on liver cancer.

Model	Capsaicin dosage	Main functions	Reference
HepG2 cells	0,25,50,75,100 and 200 μM	↓Bcl-2; Bax/Bcl-2 ratio	Chen et al. (2018)
Trpv1 null (Trpv1^−/−^) mice, wide type C57BL/6 (Trpv1^+/+^) mice, and NOD/SCID female mice	2 mg/kg	↓AFP and Ki67	Xie et al. (2019)
Western-type diet in mice	0.5 mg/kg	↓ALT and AST	Sarmiento-Machado et al. (2021)
HepG2 cells	0,50,100,200 and 250 μM	↑ROS	Lee et al. (2004)
HepG2 cells	50–250 μM	↑ROS	Baek et al. (2008)
HepG2 cells	0–800 µM	↑TOS, 8-OHdG, CASP3, CYC, Bax, and NOX4 levels; ↓Bcl-2, GSH, and SIRT1	Hacioglu (2022)
HepG2 cells	10, 50, 100 and 200 μM	↑ROS	Huang et al. (2009)
HepG2 cells	5, 10, 25, 50, 100 and 200 μM	VROS	Joung et al. (2007)
HepG2 cells	150 and 250 μM	↑intracellular Ca^2+^	Kim et al. (2005)
Hep3B and HepG2 cells	0,50,100,150,200 and 250 μM	↑DR5	Moon et al. (2012)
SK-Hep-1 cells	0,50,100,150 and 200 μM	↓Bcl-2; ↑Bax	Jung et al. (2001)
HepG2 cells	50, 100 and 200 μM	↑LC3-II and beclin-1	Chen et al. (2016)
Sprague-Dawley rats	1 mg/kg and 2 mg/kg	↓SIRT1 and SOX2	Xie et al. (2022)
LM3, Hep3B, Huh7 cells and BALB/C nude mice	LM cells: capsaicin (0.100,130,160,190,220 μM) and sorafenib (0,2,3,4,5,6,7 μM); Hep3B cells: capsaicin (0,8,16,32,64,128 μM) and sorafenib (0,0.25,0.5,1,2,3,4 μM); Huh7 cells: capsaicin (0,25,50,75,100,125) μM and sorafenib (0,0.5,0.75,1,1.25,1.5,1.75 μM); mice:5 mg/kg capsaicin and 50 mg/kg sorafenib	↓EGFR	Dai et al. (2018)
PLC/PRF/5, Huh7, and HepG2 cells	capsaicin (0, 50, 100, 150, 200, and 250 μM) and sorafenib (0, 0.3, 1, 3, 10, and 30 μM)	↑Bax; ↓Bcl-2	Zhang et al. (2018)
HepG2, Huh-7 cells and nu/nu mice	HepG2 cells: capsaicin (0,20,40,75,150,200 μM) and sorafenib (0,1,1.5,2,2.5,3 μM); Huh7 cells: capsaicin (0,10,20,40,80,100 μM) and sorafenib (0,0.2,0.4,0.75,1.5,3 μM)	↑caspase-9 and PARP	Bort et al. (2017)

### 6.2 Roles of capsaicin in the treatment of liver cancer

#### 6.2.1 Specific effects of capsaicin on TRPV1

Capsaicin is a natural bioactive compound that activates TRPV1 (Abdillah and Yun, 2024). TRPV1 is a Ca^2+^ permeable cation channel and serves as the primary heat and capsaicin sensor in humans (Kwon et al., 2021). Capsaicin, in combination with a static magnetic field (SMF), synergistically inhibits the growth of HepG2 through the mitochondria-dependent apoptosis pathway. SMF significantly enhanced the inhibitory effect of capsaicin on cancer cells. The mechanism was that SMF enhances the inhibitory effect of capsaicin on cancer cells by inducing conformational changes in the TRPV1 ion channel (Chen et al., 2018). *In vivo* studies have shown that treating tumor-bearing mice with capsaicin significantly reduces tumor volume and improves overall survival rate. In addition, TRPV1 expression is increased in capsaicin-treated mice, while alpha-fetoprotein (AFP) and Ki67 expression are decreased (Xie et al., 2019). Preventive capsaicin dietary (specifically 0.02%) mitigates carcinogenic liver damage and the development of pretumor lesions. Capsaicin reduces diethylnitrosamine (DEN)-induced oxidative damage by improving the glutathione (GSH) axis, and reducing hepatocyte necrosis and inflammation (Sarmiento-Machado et al., 2021).

#### 6.2.2 Oxidative stress

Oxidative stress is a condition where the oxidative and anti-oxidant effects in the body is disrupted. It has become a key factor in the initiation and progression of many diseases, including liver cancer (Tang et al., 2022; Li Z. et al., 2023). ROS are the most prevalent reactive chemical involved in oxidative stress during disease progression. Oxidative stress plays a unique role in the development of HCC, with excessive ROS generation being common in liver diseases of various etiologies (Liu et al., 2023). NADPH oxidase-mediated ROS production plays an important role in the mechanism of capsaicin-induced apoptosis (Lee et al., 2004). Capsaicin increases ROS production in HepG2 cells (Baek et al., 2008). The increase in total oxidant status (TOS) level and the decrease in GSH level indicate that capsaicin induces oxidative stress. The levels of 8‐hydroxydeoxyguanosine (8‐OHdG) levels are significantly increased in capsaicin-treated HepG2 and HL-7702 cells (Hacioglu, 2022). Capsaicin may covalently bind to NAD(P)H:quinone oxidoreductase (NQO1), thereby inhibiting its activity and leading to ROS production. Furthermore, p-Akt is activated, which increases the nuclear translocation of Nrf2, enhances the binding of ARE, and upregulates the expression of heme oxygenase-1 (HO-1) (Joung et al., 2007).

#### 6.2.3 Cell proliferation, apoptosis and survival

Malignant cells are characterized by abnormal signaling pathways involved in proliferation, apoptosis and angiogenesis (Davis et al., 2010). Capsaicin inhibits cell proliferation and induced apoptosis of HepG2 cells through the downregulation of Bcl-2 and the activation of pro-apoptotic molecules caspase-3 and p53 (Baek et al., 2008). Capsaicin induces apoptosis by promoting the expression of Bax, and decreasing Bcl-2 and increasing caspase-3 activation in HepG2 cells (Huang et al., 2009). Capsaicin has been shown to inhibit the proliferation of HepG2 cell. As an epigenetic marker, the expression of miR-126 is upregulated and the expression of piR-Hep-1 is downregulated after treatment. Additionally, capsaicin treatment leads to a decrease in the expression of Ki-67, phosphoinositide 3-kinase (PI3K), and mTOR, while increasing the expression of non-phosphorylated AKT. This indicates that capsaicin exerts both genetic and epigenetic effects on cell proliferation. Furthermore, capsaicin affects carcinogenesis by modulating the expression of miR-126 and piR-Hep-1 in different ways (Ates et al., 2022). Pepper fruit extracts have been found to alter the anti-oxidant capacity ofHepG2 cell lines, enhancing catalase activity and reducing the activity of NADPH-producing enzymes (Rodríguez-Ruiz et al., 2023). Activation of the Phospholipase C and the release of intracellular Ca^2+^ from inositol 1,4,5-trisphosphate (IP3) sensitive stores (Kim et al., 2005). Capsaicin enhances the apoptotic effect of tumor necrosis factor-related apoptosis-inducing ligand (TRAIL) on various cancer cells by inducing the expression of TRAIL receptor DR5 on the cell surface throughSp1 promoter activation. These findings suggest that capsaicin upregulates DR5 via calcium inflow-dependent Sp1 activation, thereby sensitizing HCC cells to TRAIL-mediated apoptosis (Moon et al., 2012). The inhibitory effect of capsaicin on SK-Hep-1 cells is primarily due to apoptosis induced by DNA fragmentation and nuclear aggregation. In addition, capsaicin effectively induces the apoptosis of SK-Hep-1 cells through a caspase-3-dependent mechanism (Jung et al., 2001). Capsaicin induces autophagy and apoptosis in HCC cells. The ROS-STAT3 pathway is involved in capsaicin-induced autophagy of HCC cells, and inhibition of autophagy enhances capsaicin’s effects in HCC cells (Chen et al., 2016). Sirtuin 1 (SIRT1) is overexpressed in liver cancer and acts as a tumor promoter through deacetylation by sex-determining region Y-box 2 (SOX2). Capsaicin treatment downregulates SIRT1, resulting in reduced deacetylation and degradation of SOX2. These results indicate that capsaicin inhibits liver cancer progression through the SIRT1/SOX2 signaling pathway (Xie et al., 2022).

#### 6.2.4 Interaction of capsaicin with sorafenib or 5-FU

Sorafenib is an oral kinase inhibitor known for its ability to inhibit tumor cell proliferation and angiogenesis and while inducing apoptosis in cancer cells, thereby improving survival rates for patients with advanced HCC (Kong et al., 2021). Capsaicin or sorafenib alone could inhibit cell proliferation and induce apoptosis (Dai et al., 2018; Zhang et al., 2018). Notably, capsaicin and sorafenib have shown synergistic effects in inhibiting the growth, invasion and metastasis of liver cancer cells, as well as enhancing cell apoptosis (Dai et al., 2018) (Zhang et al., 2018). And intratumoral injection of capsaicin did not cause significant severe toxicity (Zhang et al., 2018). Sorafenib combined with capsaicin demonstrated an enhanced anti-cancer effect. Sorafenib induced AKT activation, which led to drug resistance, whereas capsaicin’s inhibition of AKT might sensitize cells to sorafenib therapy (Bort et al., 2017). Additionally, 5-Fluorouracil (5-FU) is a widely used chemotherapy agent for various cancers (Shi et al., 2023). Capsaicin has been found to enhance the activity of anti-cancer drugs when used in combination. Capsaicin significantly enhanced the drug sensitivity of QBC939 to 5-FU. In addition, the combination of capsaicin and 5-FU demonstrated a synergistic effect in cholangiocarcinoma (CCA) xenografts, with the combined therapy yielding greater inhibition than 5-FU alone. Further research found that capsaicin inhibited 5-FU-induced autophagy in CCA cells by activating the PI3K/AKT/mTOR pathway (Hong et al., 2015).

## 7 Conclusion and perspective

This study found that capsaicin has numerous beneficial effects, including protection against liver damage, anti-diabetes, anti-obesity, anti-liver fibrosis, anti-liver cancer, pain relief and anti-oxidant. The studies mentioned indicate limited clinical studies on fatty liver. Most basic studies and a few clinical studies have shown that capsaicin improves liver inflammation and fat infiltration through mechanisms mediated by TRPV1 or independent pathways, preventing the progression of fatty liver, and providing liver protection effect. Capsaicin also improves systemic metabolic issues, including blood lipid, blood sugar, and insulin resistance. Geographically, fatty liver disease is prevalence across China, with higher rates in northern regions compared to the southern and southwestern regions (Yip et al., 2023). Moderate spicy eating may benefit fatty liver, be safe, and potentially reduce mortality. In another study on HCC incidence, patients from two hospitals in western China (Chongqing) and eastern China (Shanghai) were examined, revealing a higher incidence of HCC in eastern China. Epidemiological studies have shown that unhealthy diet, living environment and multiple carcinogenic factors may explain the regional differences in HCC incidence (Liao et al., 2017).

The capsaicin content varies among chili pepper varieties, with some being very hot and others less so or even non-spicy. An intake of 2.56 mg of capsaicin induces satiety, equivalent to 1–2 g of chili pepper. An intake of 5 mg improves blood sugar metabolism, equivalent to 2–4 g of chili pepper (Janssens et al., 2014). Clinical studies have shown that in pregnant women with gestational diabetes, taking 5 mg of capsaicin per day for 4 weeks, without changes in food calories or composition, improved blood sugar control and insulin resistance, and reduced the birth rate of larger-than-gestational-age infants (Yuan et al., 2016). Cancer-related fatigue is common symptom among Cancer patients, and exercise is a treatment (Li J. et al., 2023). Studies have found that capsaicin reduces serum lactate, ammonia, BUN (blood urea nitrogen) and creatine kinase (CK) levels, reduces physical fatigue and improves exercise performance in mice (Hsu et al., 2016). Studies have found that the 8% capsaicin patch appears to be effective in the short and medium term for treating peripheral neuropathic pain, as it not only reduces pain intensity but also decreases the pain area. Most patients tolerate its application well (Goncalves et al., 2020). Moreover, in Europe, capsaicin patches (179 mg) have been approved for the local treatment of peripheral neuropathic pain, either as a monotherapy or in combination with other medications (Maihöfner et al., 2021).

Moreover, whole chili peppers or other capsaicin-containing foods have an effect on liver health. Research indicates that dietary preferences in China vary geographically, influenced by local climate and consumption levels. Spicy regions are mainly in the southwest, centered around Sichuan province, which also has lower diabetes prevalence, possibly due to capsaicin, the main spicy ingredient in chili peppers (Zhao et al., 2020). A 2021 study showed that compared to those who do not eat spicy food, individuals who consume spicy food have reduced risks of esophageal, stomach, and colorectal cancers. The benefits are greater among non-drinkers and non-smokers (Chan et al., 2021). Furthermore, compared to individuals who consume spicy foods less than once a week, even a modest intake of spicy foods-just 1–2 days per week-has been associated with observable health benefits. Notably, consuming spicy foods 6 to 7 times per week is linked to a 14% reduction in all-cause mortality and a 22% reduction in ischemic heart disease-related mortality (Lv et al., 2015). A foreign cohort study with 22,000 participants followed for 8.2 years showed that regular chili pepper consumption reduced all-cause mortality by 23% and cardiovascular event mortality by 34% (Bonaccio et al., 2019). Another cohort study with over 50,000 people found that weekly chili pepper consumption reduced high blood pressure incidence by 28% among non-drinkers (Wang et al., 2021). A meta-study has found that eating chili peppers reduces the risk of death, potentially due to capsaicin promoting fat metabolism, increasing energy expenditure, and controlling blood sugar, thereby reducing obesity and metabolic syndrome risks, and cardiovascular disease mortality (Kaur et al., 2021). Regular chili pepper consumption (at least once a week) was shown to reduce all-cause mortality by 12% and cardiovascular event mortality by 18% (Ofori-Asenso et al., 2021). Moreover, Defatted pepper seed ethanolic extract (DPSE) reduces HFD-induced weight gain and liver cholesterol content (Sung et al., 2016). The study found that the consumption of black pepper or chili is significantly associated with a reduced risk of overall mortality (Hashemian et al., 2019). Additionally, the study also found that green Capsicum annuum exhibits hepatoprotective effects (Das et al., 2018).

While capsaicin has demonstrated significant anti-cancer potential in various preclinical models, its translation into clinical application presents several key challenges, particularly in terms of dosage determination and safety. Firstly, establishing a dosage that is both effective and safe poses a substantial challenge. The effective doses observed in animal models may not be directly applicable to humans due to differences in metabolism and toxicity responses across species. Therefore, extensive dose-escalation studies are necessary to identify an appropriate therapeutic range. Secondly, the safety profile of capsaicin cannot be overlooked. High doses of capsaicin may cause adverse effects such as gastrointestinal cramps, stomach pain, nausea, diarrhea, vomiting, increased circulating blood volume, heart rate, tachycardia and stomach cancer risk (López-Carrillo et al., 2003; Merritt et al., 2022). Some studies suggest capsaicin may also be a carcinogen, promoting cancer metastasis (Cheng et al., 2023a; Deng et al., 2023; Cheng et al., 2023b). Despite its anti-cancer activity, capsaicin’s clinical use as an anti-cancer drug remains problematic due to poor bioavailability and water solubility (Giri et al., 2016). Furthermore, the delivery method of capsaicin is another significant challenge. While local delivery may help mitigate systemic toxicity, ensuring sufficient concentration at the tumor site without causing widespread adverse effects remains a critical area for further research, especially in the treatment of systemic cancers (Giri et al., 2016; Lu et al., 2020). Therefore, patients with liver disease are advised to consume spicy food in moderation to satisfy appetite without aggravating their condition.

Research on chronic liver diseases has increasingly focused on fatty liver disease, particularly NAFLD, due to its close association with metabolic syndrome. Studies have shown that capsaicin consumption may have beneficial effects, such as improving cardiovascular outcomes and reducing all-cause mortality, which is particularly relevant in the context of fatty liver disease. Given these findings, the long-term effects of capsaicin on chronic liver diseases, especially metabolic-related fatty liver, could be a promising area for future research. However, more studies are needed to fully understand its impact compared to other liver conditions, such as hepatic injury or HCC. Future research could focus on determining the optimal dosage and safety profile of capsaicin for clinical use, particularly in the treatment of liver diseases and cancers, while also exploring the mechanisms through which capsaicin exerts its protective effects on liver health and its potential impact on systemic metabolic issues. Additionally, investigating regional dietary habits in China, especially the varying impacts of capsaicin consumption on health outcomes, could provide valuable insights. Exploring novel delivery methods for capsaicin to improve its bioavailability and minimize adverse effects represents another crucial area for future investigation.
